# RETRACTED: Zhou et al. HOXA11-AS1 Promotes PD-L1-Mediated Immune Escape and Metastasis of Hypopharyngeal Carcinoma by Facilitating PTBP1 and FOSL1 Association. *Cancers* 2022, *14*, 3694

**DOI:** 10.3390/cancers17121917

**Published:** 2025-06-09

**Authors:** Zheng Zhou, Qian Liu, Gehou Zhang, Diab Mohammed, Sani Amadou, Guolin Tan, Xiaowei Zhang

**Affiliations:** 1 Department of Otolaryngology Head & Neck, Third Xiangya Hospital, Changsha 410013, China; 2 Department of Otolaryngology Head & Neck, Xiangya Hospital, Changsha 410008, China; 3 Department of ENT, Reference Hospital, Maradi 12481, Niger

The journal retracts the article “HOXA11-AS1 promotes PD-L1–mediated immune escape and metastasis of hypopharyngeal carcinoma by facilitating PTBP1 and FOSL1 association” [1], cited above.

Following publication, concerns were brought to the attention of the Editorial Office regarding image duplication across several publications produced by different authorships.

Adhering to our standard procedure, an investigation was conducted by the Editorial Office and the Editorial Board that confirmed the duplication reported between Figure 6I published in [1] and Figures 4G, 6G and 8B in [2], as well as Figure 7G in [3], presenting different experimental conditions. Given the extent of the duplications, the Editorial Board has lost confidence in the reliability of the findings and decided to retract this publication [1], as per MDPI’s retraction policy (https://www.mdpi.com/ethics#_bookmark3).

This retraction was approved by the Editor-in-Chief of the *Cancers* journal.

Xiaowei Zhang agrees to this retraction. The remaining authors did not provide a comment on this decision.
